# Supplementary material for: DNA sliding and loop formation by E. coli SMC complex: MukBEF
Source: Biochem Biophys Rep. 2022 Jun 22;31:101297. doi: 10.1016/j.bbrep.2022.101297 (PMC9234588; doi:10.1016/j.bbrep.2022.101297)
Supplement: Multimedia component 1 [file mmc1.docx]

**Supplementary figures:**


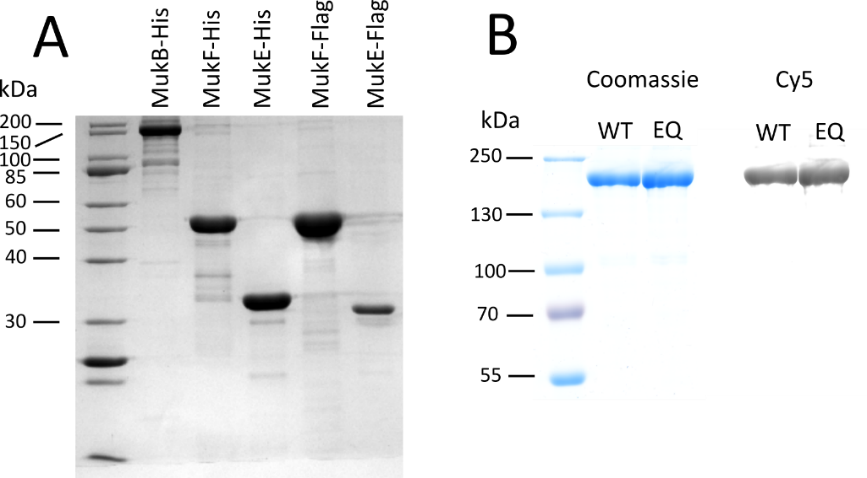


**Fig S1.** (A) Coomassie blue staining of purified proteins. (B) The purified and labeled Wild-type MukB (WT) and ATP hydrolysis-defective mutant MukB^EQ^ (EQ) were analysed by SDS-PAGE followed by Coomassie blue staining and in gel fluorescence detection of Cy5 dye. The labeling efficiency of homodimeric MukB is ~ 65%.


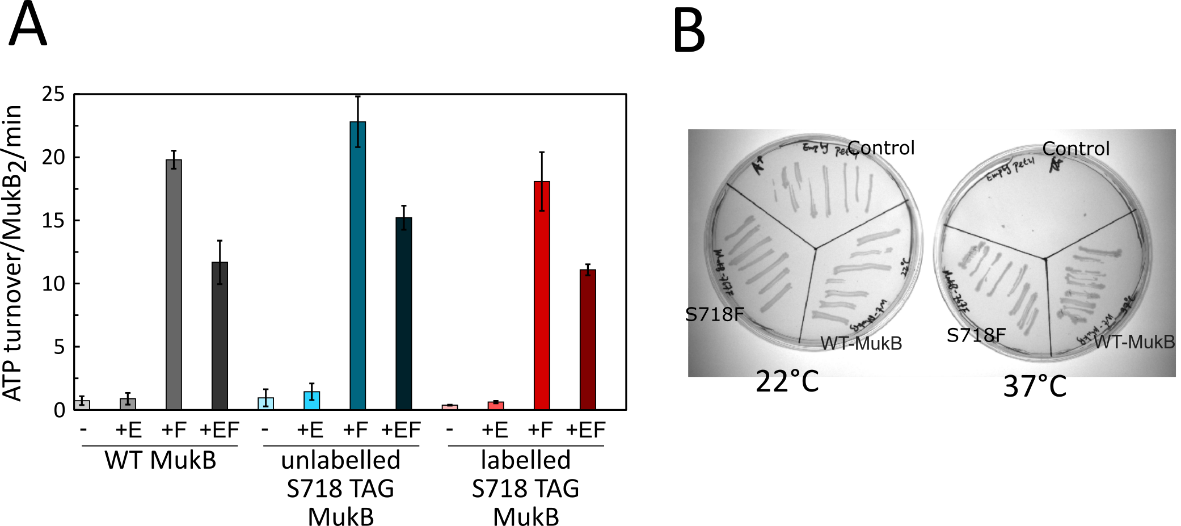


**Fig S2.** (A)The ATPase activities (Mean ± SEM) of MukB, S718TAG MukB, and Cy5(Cy3) labelled MukB, in the presence of the indicated components. (B) Functional analysis by complementation assay of mutated MukB_S718S. Cells carrying MukB_S718F and cells carrying WT-MukB were compared at permissive (22°C) and non-permissive (37°C). Control: empty vector pET-21(+) as the negative control. The MukB_S718F fully complements the temperature-sensitive growth defect of ∆mukB cells in vivo.


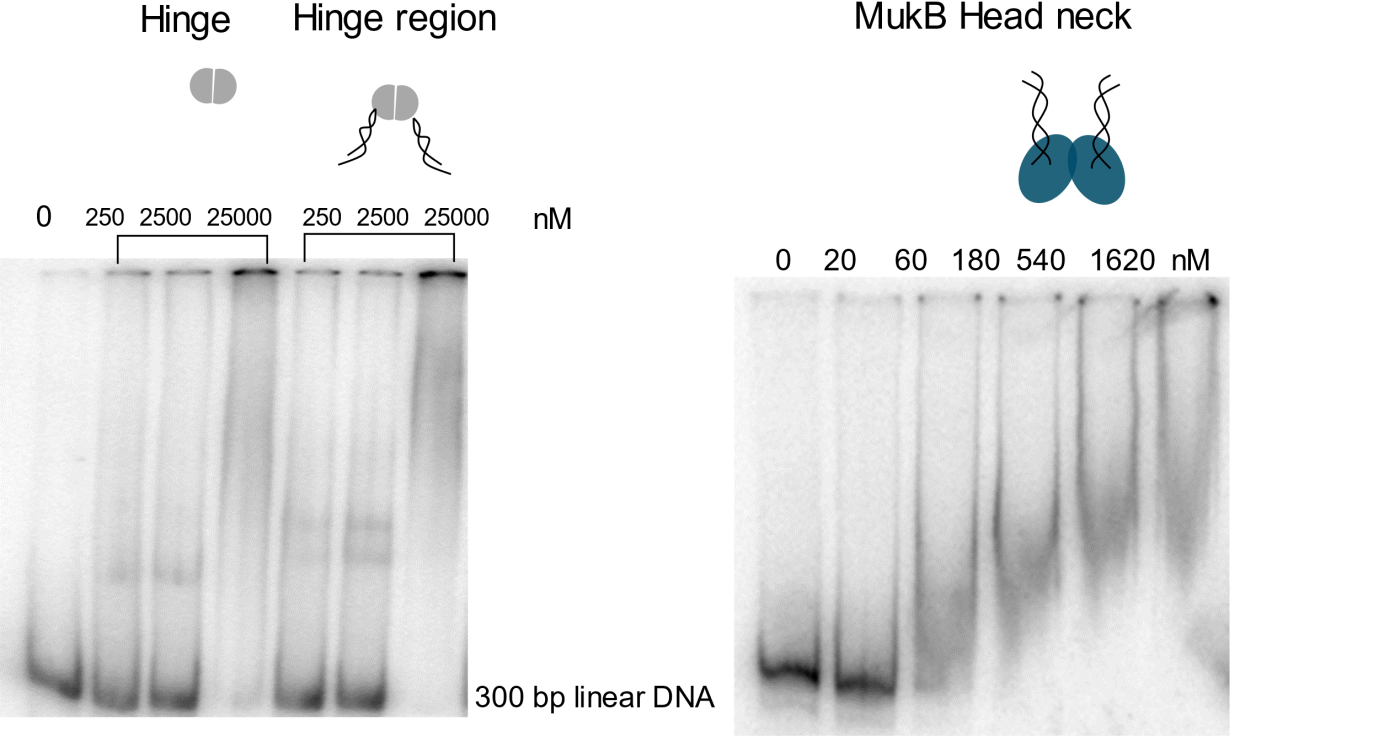


**Fig. S3. Gels from DNA electrophoretic mobility shift assay (****EMSA) to test binding affinity between MukB fragments and DNA *in vitro*.** The DNA fragment concentration was 300 nM. Hinge of MukB and Hinge range of MukB concentrations were: 250, 2500 and 25000 nM, respectively. MukB head neck concentration were: 20, 60, 180, 540, 1620 nM. Samples were incubated for 30 min at room temperature in T50 buffer and applied to a 6 % polyacrylamide gel equilibrated with the same buffer. Electrophoresis was at 120 V for 1 h. Concentration “0” denotes free DNA. Very weak binding between DNA and MukB fragments can be observed.


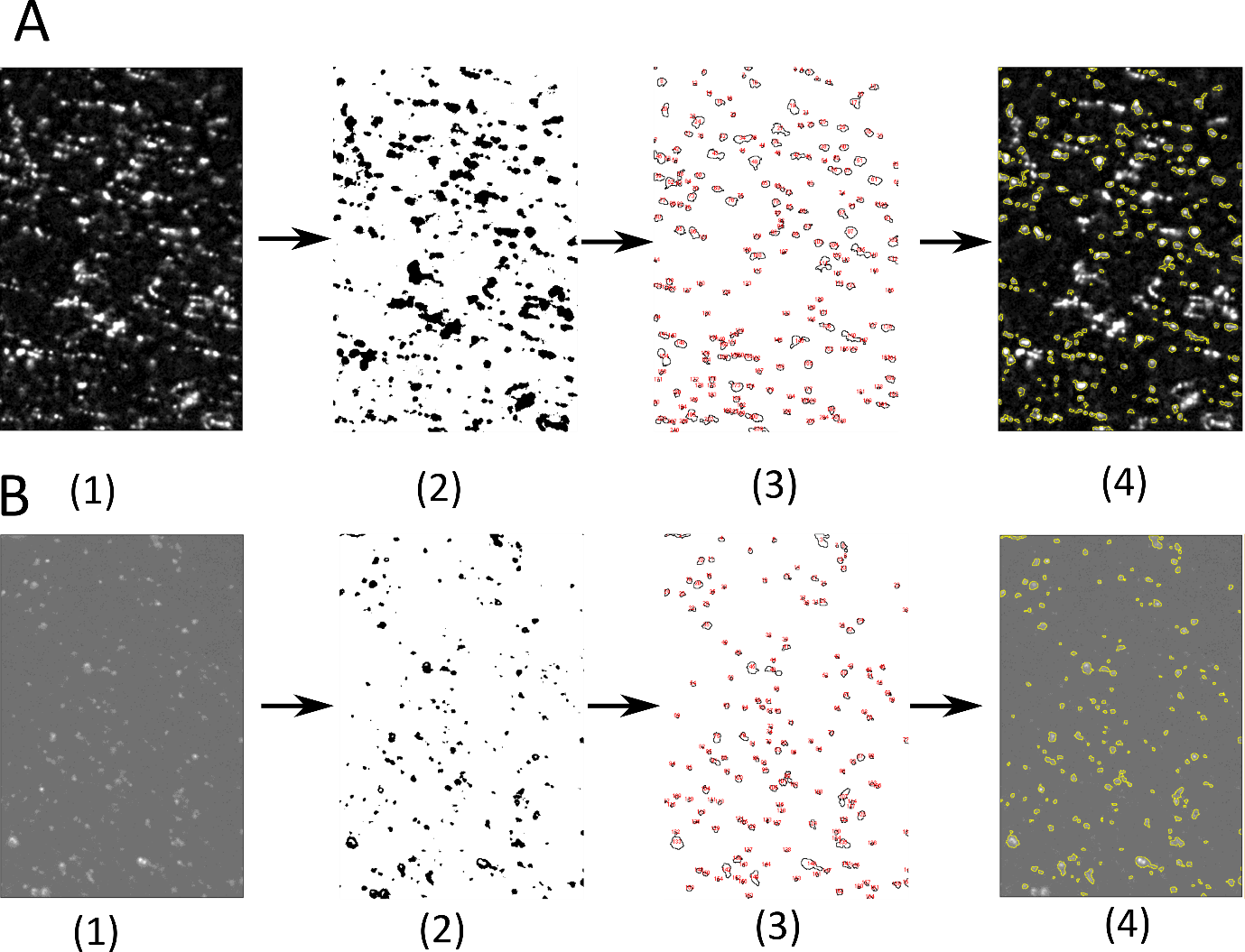


**Fig S4. Example of imaging process to quantify intensity of MukB^EQ^ clusters with MukE and MukF (A) or MonoMukF (B) on the tethered DNA.** (1) The image after subtracting background (2) A binary mask after applying thresholding (3) Identified ranges by applying ‘analyse particles’ on the mask, that were between 9-300 pixels and with the circularity between 0.2 and 1. (4) Overlapped ranges with the original image.


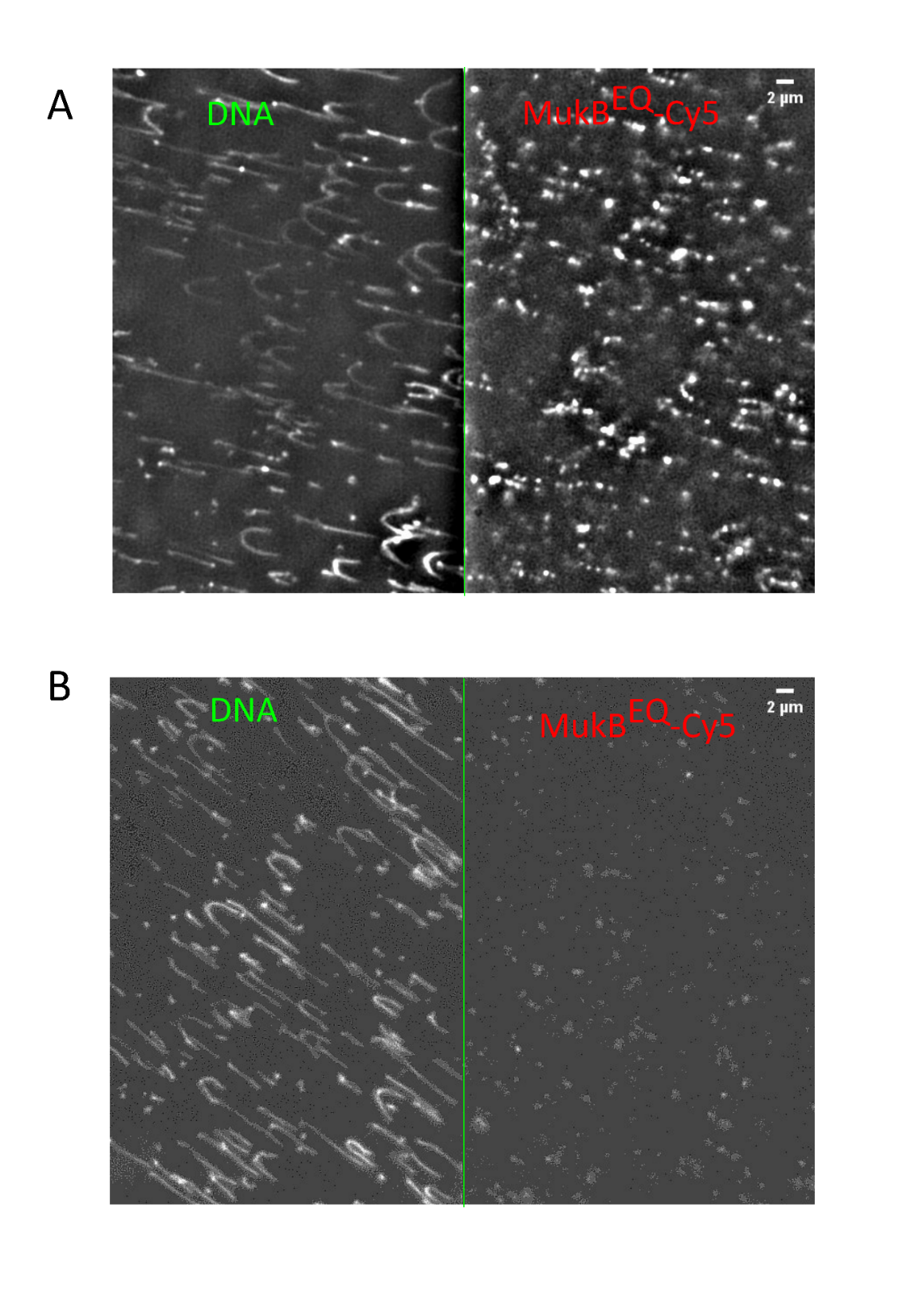


**Fig. S5 (A).** Snap-shot of MukB^EQ^ EF clusters with ATP-Mg^2+^ in 150mM NaCl under 1 pN force. **(B).** Snap-shot of MukB^EQ^ with MukE, MonoMukF and ATP-Mg^2+^ in 150mM NaCl on the biotinylated anti-His_6_-antibody functionalized PEG surface. Left channel is Sytox Orange stained DNA, and the right channel is Cy5 labelled MukB^EQ^.


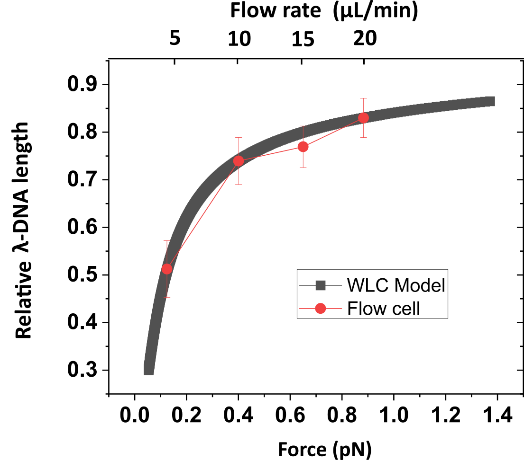


**Fig. S6 Calibration of applied force and flowrate.** The flowrate is between 1ul/min to 100ul/min. The applied force is calibrated based on the previous studies (*69*). The relationship between applied flow rate and relative λDNA length, fitted to the Worm-Like Chain (WLC) model (solid line) with the fitted persistence length of 43 nm and a contour length of 16.3 μm.
