# Supplementary material for: DNA sliding and loop formation by E. coli SMC complex: MukBEF
Source: Biochem Biophys Rep. 2022 Jun 22;31:101297. doi: 10.1016/j.bbrep.2022.101297 (PMC9234588; doi:10.1016/j.bbrep.2022.101297)
Supplement: Multimedia component 2 [file mmc2.docx]

**Supplementary movies**

1. Movie showing fluctuation of doubly tethered λDNA incubated with 10 nM MukB, 20 nM MukE, 10 nM MukF, and 1 mM ATP-Mg^2+^. Scale bar, 2 µm.
2. Movie showing fluctuation of doubly tethered λDNA incubated with 10 nM MukB-Cy5, 20 nM MukE, 10 nM MukF, and 1 mM ATP-Mg^2+^. Scale bar, 2 µm. The 640 nm laser is switched on after 30 min incubation of MukBEF, ATP-Mg^2+^ with DNA. Image is split into two channels: the left one is the red channel to visualize Cy5 labelled MukB, and the right one is the green channel to visualize Sytox orange-stained DNA.
3. Movie showing compaction of doubly tethered λDNA by 10 nM MukB. Locally compacted DNA (DNA ‘bulbs’) can be observed on λDNA. It takes ~ 1000 s until DNA is fully compacted.
4. Movie showing compaction of doubly tethered λDNA by 100 nM MukB. Locally compacted DNA (DNA ‘bulbs’) can be observed on λDNA. It takes ~ 150 s until DNA is fully compacted.
5. Movie showing distribution of MukB-Cy5 on the over-stretched the DNA. Image is split into two channels: the left one is the red channel to visualize Cy5 labelled MukB, and the right one is the green channel to visualize Sytox orange-stained DNA.
6. Movie showing fluctuation of doubly tethered λDNA and no DNA compaction with 100 nM MukB and 1mM ATP-Mg^2+^. Incubation for 30 min, then switch on the 640 nm laser. Image is split into two channels: the left one is the red channel to visualize Cy5 labelled MukB, and the right one is the green channel to visualize Sytox orange-stained DNA.
7. Movie showing one example of DNA compaction. DNA ‘bulbs’ can be observed on λDNA by either 20 nM MukB^EQ^, or 20 nM MukB^EQ^ with 1mM ATP, or 100 nM MukB^DA^, or 100 nM MukB^DA^ with 1mM ATP.
8. Movie showing MukBEF cluster with ATP-Mg^2+^ on the DNA ‘bulb’ (we noticed that when there is ‘blub’ in the DNA channel (green channel), the emission of Sytox orange leaking to the red channel is significant and this makes single MukB detection challenging.
9. Movie showing DNA loop formed with MukBEF cluster and ATP-Mg^2+^ under

~ 1  pN of applied force. The loop does not grow over time.

1. Movie showing MukB^EQ^ EF clusters with ATP-Mg^2+^ on the DNA at 150 mM NaCl and ~1 pN of applied force. Image is split into two channels: the left one is the red channel to visualize Cy5 labelled MukB^EQ^, and the right one is the green channel to visualize Sytox orange-stained DNA.
2. Movie showing one example of DNA loop formed by MukB^EQ^ EF cluster with ATP-Mg^2+^ at 150 mM NaCl and ~1 pN of applied force. DNA channel (green) is overlapped with Cy5-MukB^EQ^ channel (red).
3. Movie showing MukB^EQ^ MukE and MonoMukF with ATP-Mg^2+^ on the DNA at 150 mM NaCl and ~1 pN of applied force. Image is split into two channels: the left one is the red channel to visualize Cy5-MukB^EQ^, and the right one is the green channel to visualize Sytox orange-stained DNA.
4. Movie showing plasmid diffusion in the solution on the immobilized MukB surface, after plasmid was incubated with 10 nM MukB-His, 10 nM MukE-Flag + 20 nM MukF-Flag and 1mM ATP. DNA channel (green) is overlapped with Cy5-MukB channel (red).
5. Movie showing captured circular DNA (plasmid) on the immobilized MukB surface, after plasmid was incubated with 10 nM MukB-His. DNA channel (green) is overlapped with Cy5-MukB channel (red).
6. Movie showing a circular plasmid DNA is stretched on the immobilized MukB at two points. DNA breaks, slides through MukB, and sequentially releases from the surface.
7. Movie showing λDNA is stretched on the immobilized MukB surface with two ends spinning. DNA breaks, slides through MukB, and releases from the surface.
8. Movie showing a circular λDNA is stretched on the immobilized MukB at four points. DNA breaks, slides through MukB, and sequentially releases from the surface.
9. Movie showing stretched λDNA on the immobilized MukB surface and washed with MukE-Flag, MukF-Flag and 1mM ATP-Mg^2+^ in 150 mM NaCl.
10. Movie showing stretched λDNA on the immobilized MukB surface and washed with MukE-Flag, MukF-Flag and 1mM ATP-Ca^2+^ in 150 mM NaCl. Afterwards, the laser power was increased to break λDNA.
11. Proposed model based on published crystal structure: DNA topologically entrapped round MukB head with C terminus of MukF.
